# Innovative nomogram for predicting 1-year clinically driven revascularization outcomes in endovascular femoropopliteal disease

**DOI:** 10.3389/fcvm.2024.1438214

**Published:** 2024-08-28

**Authors:** Dikang Pan, Sensen Wu, Hui Wang, Yachan Ning, Jianming Guo, Cong Wang, Lianrui Guo, Hongfei Sang, Yongquan Gu

**Affiliations:** ^1^ Vascular Department, Xuanwu Hospital, Capital Medical University, Beijing, China; ^2^ Intensive Care Unit, Xuanwu Hospital, Capital Medical University, Beijing, China; ^3^ Vascular Surgery, The Second Affiliated Hospital of Soochow University, Suzhou, China

**Keywords:** nomogram, target revascularization, risk factor, atherosclerosis, femoropopliteal artery disease

## Abstract

**Purpose:**

Femoropopliteal artery disease (FPAD) is a common vascular disease that usually requires surgical treatment. The aim of this study was to apply predictive modeling to develop predictive models for predicting clinically driven target revascularization (CD-TLR) events 1 year after intervention in patients with FPAD.

**Materials and methods:**

In this study, clinical data were collected from a total of 484 patients who underwent FPAD endovascular intervention from 2014 to 2019. According to the inclusion and exclusion criteria, 400 patients will be finally included and assigned to the training cohort and test cohort in the ratio of 7:3. By analyzing these data through statistical methods, we will explore the effects of different factors on target revascularization events 1 year after intervention in FPAD patients, and build the corresponding prediction model of the column line graph.

**Results:**

The final nomogram model consisted of 5 independent predictors: history of cerebrovascular disease, lesion length >15 cm, no atherectomy device used, no medicated balloon used and procedure time. The C-index of the model was 0.766 and 0.726 for the training cohort and validation cohort, respectively. The calibration curves also showed that the model had satisfactory agreement in both cohorts.

**Conclusions:**

The newly developed prediction model can accurately predict clinically driven target revascularization events at 1 year in patients with FPAD, providing valuable information for the development of individualized treatment plans.

## Introduction

Obstructive atherosclerosis of the peripheral vascular system characterizes peripheral arterial disease (PAD), which often refers to the arteries in the lower limbs (1). Sometimes, it is considered a precursor to atherosclerosis in other blood vessels (2). Myocardial infarction, ischemic stroke, and cardiovascular mortality are more likely in those with PAD (3). It affects 3%–7% of the general population and 20% of persons over the age of 75, and the economic cost and health risks it imposes cannot be disregarded (4). The femoropopliteal artery is the most common site of PAD, which can lead to coldness and numbness of the affected limb, intermittent claudication, ulceration, and even gangrene of the limb leading to amputation (5), which can seriously affect the quality of life of patients.

Currently, endovascular therapy has become a common treatment for femoropopliteal artery disease (FPAD), including the use of stents, medicated balloons, and volume-reducing devices (3). However, clinically driven target lesion revascularization after femoropopliteal artery intervention remains a challenging problem. Several large randomized controlled studies have now found that CD-TLR rates after femoropopliteal artery interventions vary from approximately 4.9% to 26% (6–8). Therefore, accurate identification of post-interventional vascular CD-TLR in patients with FPAD is critical in the clinical setting.

A nomogram is a visual statistical prognostic tool that is widely used in the clinical evaluation of prognosis by calculating scores based on potential predictive factors (9). It can provide a quick assessment of clinical risk stratification and prognosis judgment (10). Therefore, the aim of this study is to construct a predictive model to help clinicians predict whether a patient will have a CD-TLR events within 1 year after FPAD intervention. It will provide a strong basis for clinical decision-making after femoropopliteal artery intervention and help physicians accurately assess patients' risk and take appropriate preventive measures. This will help improve patients' postoperative prognosis and enhance treatment outcomes.

## Methods

### Study design and participants

**Figure 1:**
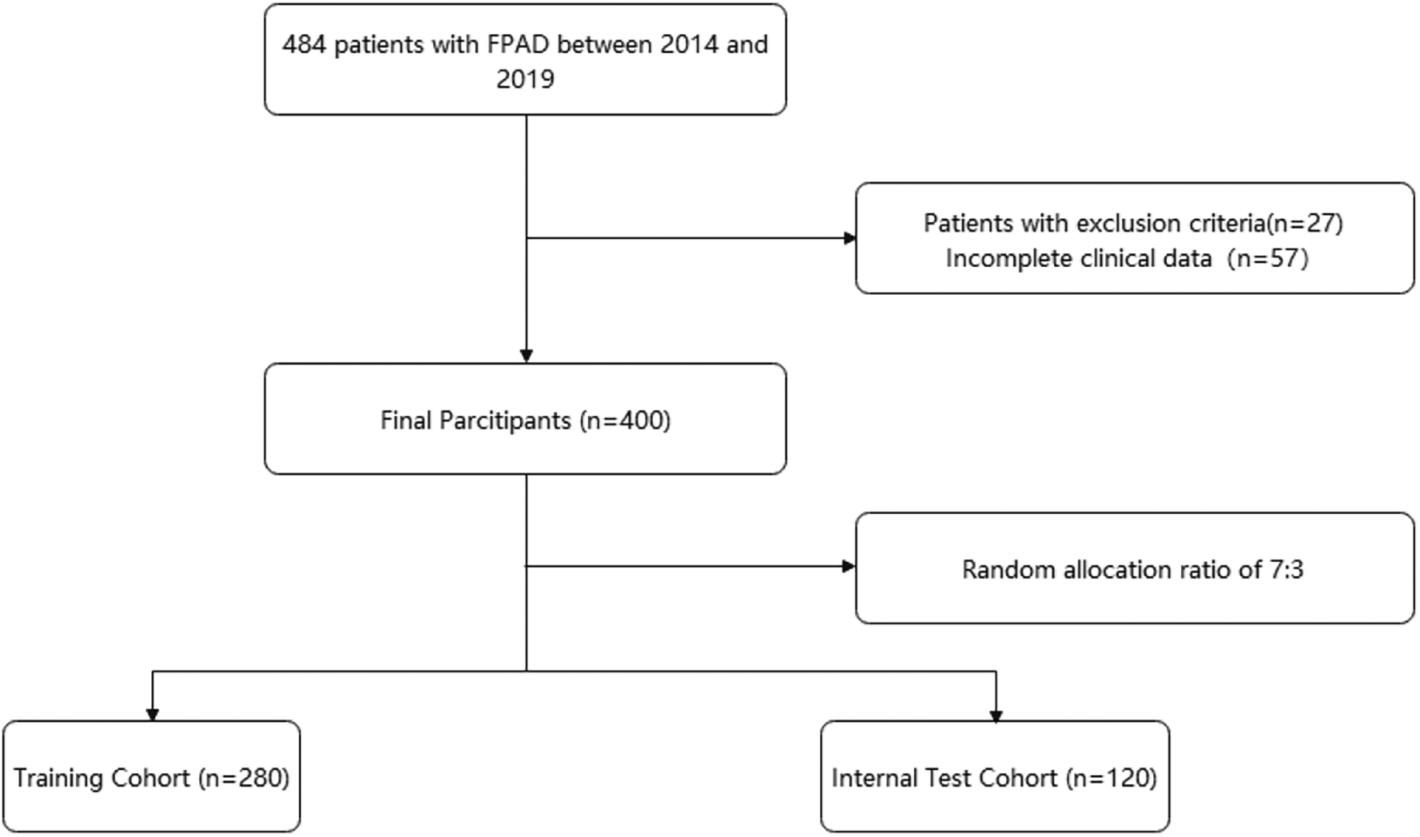
Flow chart of the training and validation cohorts.

Figure 1 shows the flow chart of our study. This retrospective study was based on an electronic medical record system for inpatients at a large medical center. Patients who underwent FPAD intervention from January 2014 to June 2019 were included in the study. Inclusion criteria: (1) target lesion was located in the femoral and popliteal arteries; (2) successful revascularization technique after admission; (3) known baseline information and post-procedure follow-up. Exclusion criteria: (1) target lesions located in the thoracic and abdominal aorta; (2) other causes of lower limb ischemia, including but not limited to traumatic arterial injury, aneurysm, etc.; (3) patients with previous malignant/active tumors. Finally, 484 patients were included. After excluding patients with incomplete clinical data or those who met exclusion criteria (*n* = 84, 17.3%), 400 patients were eligible for analysis. All patients were assigned to the training and validation sets in a 7:3 ratio.

We collected baseline medical information including age, gender, co-morbidities (hypertension, type 2 diabetes, coronary heart disease, atrial fibrillation, hyperlipidemia, cerebrovascular disease), history of smoking and alcohol consumption, length of hospitalization, duration of illness, and whether or not the patient had a CLTI (chronic limb-threatening ischemia, rutherford ≥4). Laboratory markers were obtained from hematology tests performed within 24 h of admission. Procedure-related information was also recorded, including procedure time, revascularization strategy, and equipment used. Variables with more than 20% missing data were excluded. Missing data <20% were extracted using the random forest method (11).

Endpoint events included CD-TLR occurring within 1 year. CD-TLR was defined as any re-intervention within the target lesion that was required due to clinical symptoms or a decrease in ankle body index(ABI) of ≥20% or >0.15 compared with the ABI after the initial procedure. Follow-up was performed until June 30, 2023, and patient follow-up information was recorded by telephone or outpatient interview, and time of death and time of amputation were recorded. One group of physicians collected clinical information and the other group obtained follow-up results.

### Statistical analysis

Non-normal data were presented as median (interquartile ranges). Chi-square test or Fisher's exact test was used to analyze the categorical variables, while the t-test or rank-sum test was used to examine the continuous variables. In the training cohort, the least absolute shrinkage and selection operator (LASSO) logistic regression analysis was used for multivariate analysis to screen the independent risk factors and build a prediction nomogram for CD-TLR. The performance of the nomogram was assessed using the receiver operating characteristic (ROC) curve and calibration curve, with the area under the ROC curve (AUC) ranging from 0.5 (no discriminant) to 1 (complete discriminant). A decision curve analysis (DCA) was also performed to determine the net benefit threshold of prediction. Results with a *p*-value of <0.05 were considered significant. All statistical analyses were performed using the R software (version 4.2.2).

## Results

### Baseline characteristics

The baseline characteristics of the study population are summarized in Table 1. Age was comparable between the training cohort (median: 66 years, IQR: 60–74) and the internal test cohort (median: 70 years, IQR: 63–76). There was a statistically significant difference in age between the two groups (*p* = 0.003). Gender distribution was similar, with approximately 76% males in both cohorts (*p* = 0.838). The prevalence of smokers was similar in both cohorts, with 55% and 54% non-smokers, and 45% and 46% smokers in the training and internal test cohorts, respectively (*p* = 0.826). Similarly, there were no significant differences in the prevalence of hypertension, T2DM, CAD, cerebrovascular disease, renal disease, revascularization history, cancer, CLTI, or occlusive lesion between the two cohorts (*p* > 0.05 for all). However, there were differences in blood sugar levels (*p* = 0.009), triglyceride levels (*p* = 0.001), and BMI category (*p* < 0.001) between the training and internal test cohorts. Overall, these results suggest that the two cohorts were comparable in terms of age and gender distribution, but differed in certain clinical characteristics.

**Table 1:** Patient demographics and baseline characteristics.

Characteristic	Training cohort, *N* = 280^a^	Internal test cohort, *N* = 120^a^	*p*-value^b^
Age	66 (60, 74)	70 (63, 76)	0.003
Male	212 (76%)	92 (77%)	0.838
Smoke	125 (45%)	55 (46%)	0.826
Drink	63 (23%)	23 (19%)	0.457
Hypertension	213 (76%)	81 (68%)	0.075
Type 2 diabetes	174 (62%)	68 (57%)	0.305
Coronary heart disease	83 (30%)	37 (31%)	0.812
Cerebrovascular disease	66 (24%)	25 (21%)	0.565
Renal insufficiency	12 (4.3%)	4 (3.3%)	0.786
Revascularizition history	53 (19%)	19 (16%)	0.460
Cancer	4 (1.4%)	6 (5.0%)	0.072
CLTI	66 (24%)	38 (32%)	0.091
Red blood cell	4.47 (4.12, 4.80)	4.29 (3.90, 4.71)	0.011
Hemoglobin	135 (125, 146)	132 (118, 144)	0.092
Fibrinogen	3.67 (3.19, 4.34)	3.81 (3.18, 4.59)	0.383
AST	16 (12, 22)	14 (11, 21)	0.086
Total bilirubin	10.5 (8.1, 13.5)	11.0 (7.3, 14.3)	0.959
Albumin	38.8 (36.5, 41.4)	38.5 (35.4, 41.4)	0.345
ALT	19.0 (16.0, 22.5)	20.0 (17.0, 23.0)	0.166
LDH	174 (153, 200)	174 (150, 204)	>0.999
Blood sugar	5.92 (5.03, 7.63)	5.35 (4.62, 7.01)	0.009
Total cholesterol	3.85 (3.20, 4.66)	3.84 (3.22, 4.53)	0.901
White blood cell	6.85 (5.72, 8.17)	6.56 (5.37, 8.22)	0.273
Hospital time	9.00 (8.00, 11.00)	9.00 (8.00, 11.00)	0.872
Triglyceride	1.48 (1.10, 2.12)	1.15 (0.93, 1.80)	0.001
High density lipoprotein	1.09 (0.91, 1.32)	1.13 (0.89, 1.40)	0.543
Outflow score	3.00 (0.00, 5.00)	3.00 (0.00, 6.00)	0.278
Lesion length			0.089
>15 cm	191 (68%)	92 (77%)	
≤15 cm	89 (32%)	28 (23%)	
Lesion type			0.205
Denovo	247 (87%)	111 (93%)	
In stent restenosis	33 (12%)	9 (7.5%)	
Occlusive lesion	163 (58%)	66 (55%)	0.552
Degree of calcification			0.137
Mildly	29 (10%)	14 (12%)	
Moderately	164 (59%)	57 (48%)	
Severe	85 (31%)	47 (40%)	
Low density lipoprotein	2.40 (1.79, 2.97)	2.32 (1.68, 2.95)	0.415
Stenting	170 (61%)	81 (68%)	0.198
Atherectomy	75 (27%)	23 (19%)	0.104
DCB	78 (28%)	25 (21%)	0.141
Distal protection device	56 (20%)	17 (14%)	0.166
Surgery time (min)	77 (59, 99)	81 (62, 99)	0.460
Uric acid	337 (289, 395)	310 (266, 357)	<0.001
Body mass index			<0.001
<24.0	43 (15%)	120 (100%)	
24.0–28.0	186 (66%)	0 (0%)	
>28.0	51 (18%)	0 (0%)	

^a^ *n* (%).

^b^ Wilcoxon rank sum test; Pearson's Chi-squared test; Fisher's exact test.

### Predictive model

The candidate predictors, age, gender, smoke, drink, hypertension, type 2 diabetes, cardiovascular disease, cerebrovascular disease, renal disease, revascularization history, cancer, chronic limb-threatening ischemia, red blood cell, hemoglobin, fibrinogen, aspartate aminotransferase, total bilirubin, albumin, alanine aminotransferase, lactate dehydrogenase, blood sugar, total cholesterol, white blood cell, hospital time, triglyceride, high density lipoprotein, outflow score, lesion length, *de novo* lesion, occlusive lesion, degree of calcification, low density lipoprotein, stenting, atherectomy (ATH), drug-coated balloon, distal protection device, surgery time, uric acid, and body mass index (<24.0, 24.0–28.0, >28.0), were included in the original model, which were then reduced to 5 potential predictors using LASSO regression analysis performed in the training cohort. Coefficient profiles and cross-validation error plots for the LASSO regression model are shown below (Supplementary Figures S1, S2). The most regularized and parsimonious model, with a cross-validated error within one standard error of the minimum, included 5 variables.

**Figure 2:**
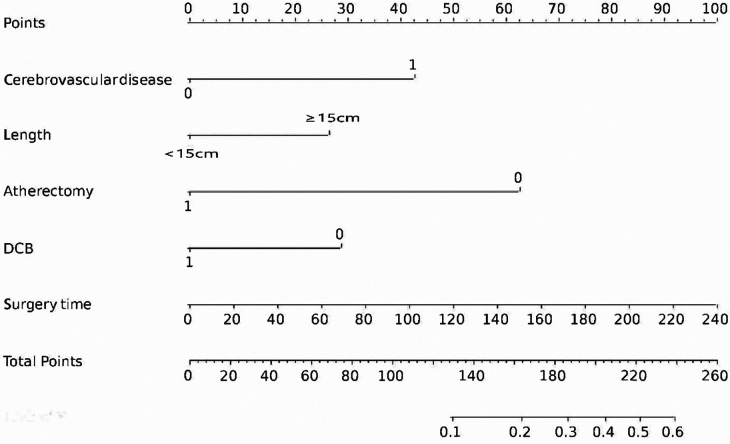
Nomogram for estimating 1-year CD-TLR. Nomogram constructed using predictors of cerebrovascular disease, DCB, ATH, lesion length, and time to surgery.

The final logistic model included 5 independent predictors (Atherectomy, DCB, cerebrovascular disease, lesion length, and surgery time) and was developed as a simple-to-use nomogram, which is illustrated in the following figure (Figure 2).

**Figure 3:**
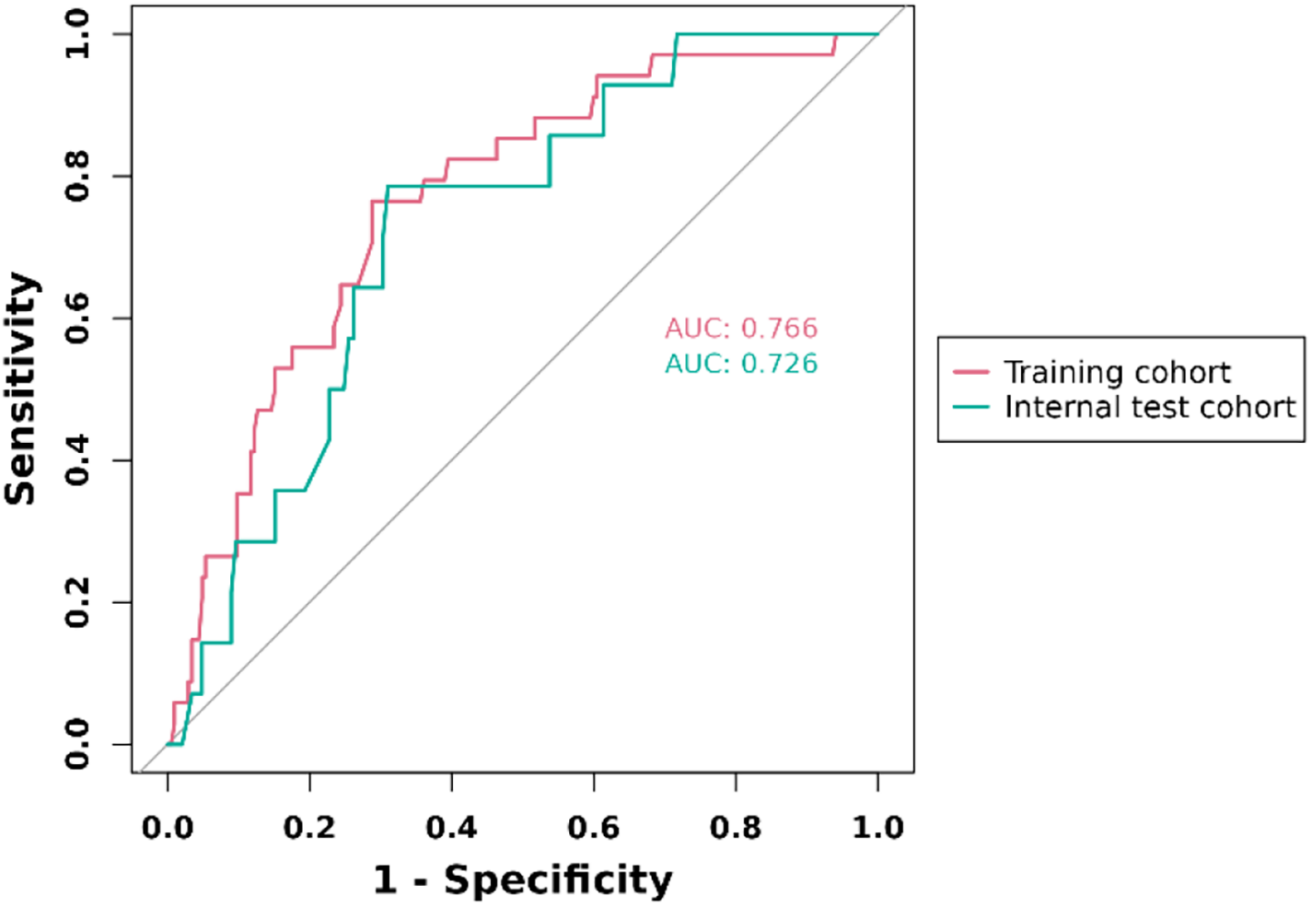
ROC curve for prediction of 1-year clinically driven target revascularization events in FPAD patients receiving endovascular therapy with the nomogram model.

ROC curves for training set and internal set were used to evaluate the discriminability of the model. the results of ROC analysis showed that the AUC of the training set was 0.766, and the AUC of the internal set was 0.726, indicating that our model has good stability and prediction accuracy. The results are displayed in Figure 3.

### Calibration curves

**Figure 4:**
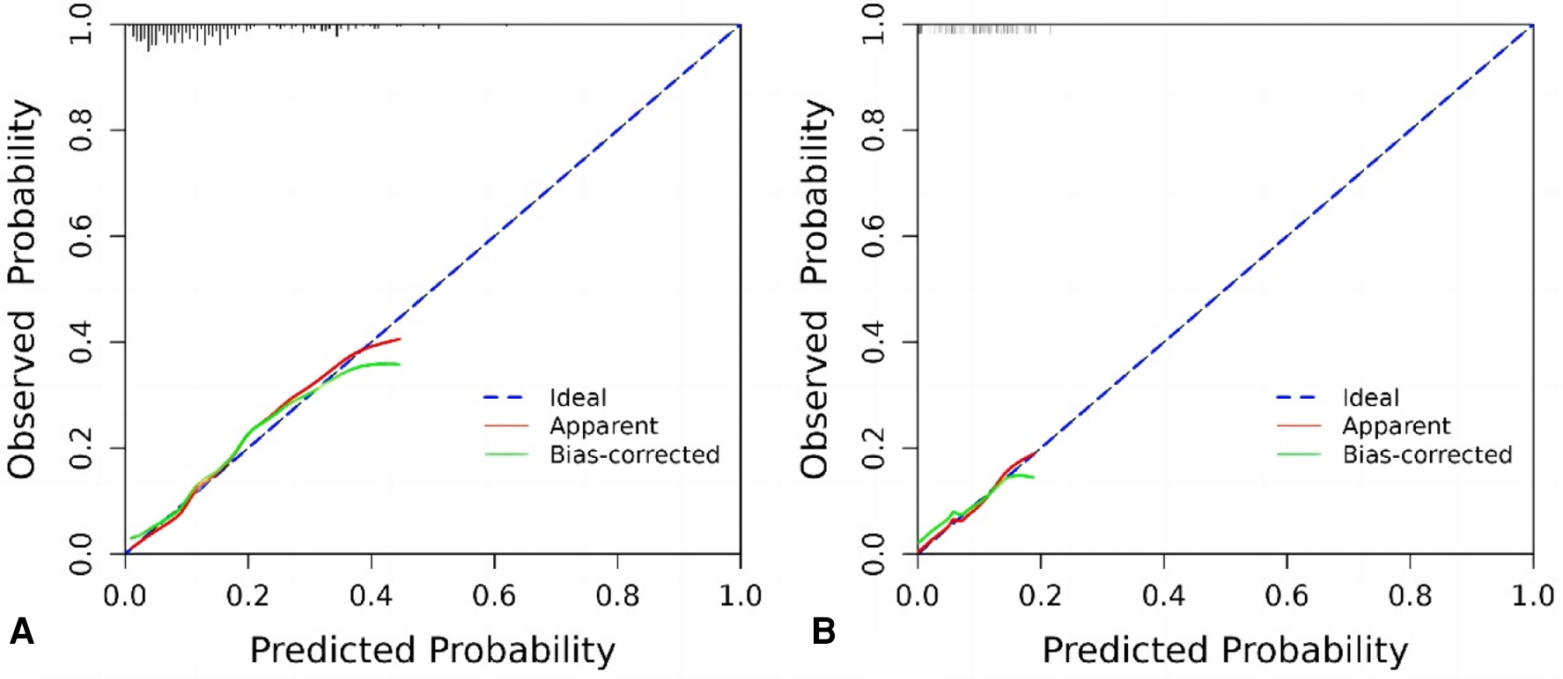
Calibration curve graph for prediction with the nomogram model. **(A)** Training cohort, **(B)** internal test cohort.

The internal validation and calibration of the nomogram were performed using 1,000 bootstrap analyses. The calibration plots of the nomogram in the different cohorts are plotted in the following figures, which demonstrate a good correlation between the observed and predicted CD-TLR. The results showed that the original nomogram was still valid for use in the validation sets, and the calibration curve of this model was relatively close to the ideal curve, which indicates that the predicted results were consistent with the actual findings (Figures 4A,B).

### Decision curve analysis

**Figure 5:**
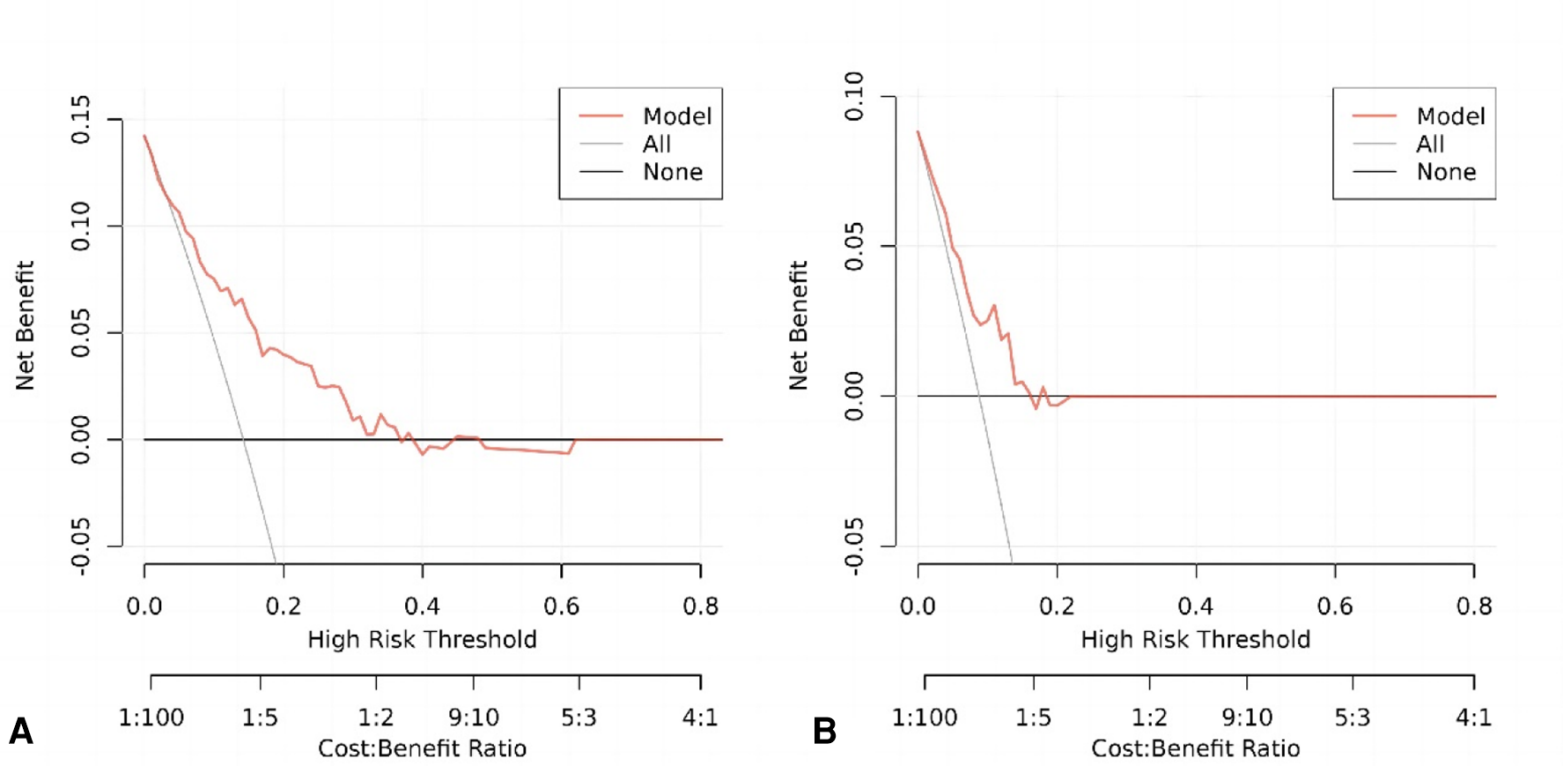
**(A)** Decision curve analysis of the nomogram of the training cohort. **(B)** Decision curve analysis of the nomogram of the internal test cohort.

The following figure displays the DCA curves related to the nomogram. A high-risk threshold probability indicates the chance of significant discrepancies in the model's prediction when clinicians encounter major flaws while utilizing the nomogram for diagnostic and decision-making purposes. This research shows that the nomogram offers substantial net benefits for clinical application through its DCA curve (Figures 5A,B).

## Discussion

In the current study, we developed and validated a nomogram for predicting 1-year CD-TLR in a cohort of patients with FPAD. The main predictors included in the nomogram were history of cerebrovascular disease, lesion length greater than 15 cm, absence of ATH, absence of DCB, and time of surgery. These predictors were found to be statistically significant in the multivariate logistic regression analysis.

Our findings are consistent with previous literature in several aspects. For instance, cerebrovascular disease has been identified as a significant predictor. It is known to be one of the leading causes of death and disability worldwide and is often a result of atherosclerosis, which is a systemic disease involving multiple vascular beds such as the brain, coronary arteries, and peripheral arteries (12). As a result, cerebrovascular disease is likely to complicate peripheral arterial disease (13). Ahn conducted a prospective study with 576 patients with cerebrovascular disease to assess the ankle-brachial index. The results showed a trend towards increased risk of PAD in patients with coronary artery disease or cerebrovascular disease, emphasizing the importance of routine ABI measurement in these high-risk groups for early detection and management of PAD (14). The REACH study, a multicenter cohort study involving 67,888 patients with multiple vascular risk factors, found a 4% increase in 3-year vascular mortality and a 7% increase in the primary endpoint (myocardial infarction, stroke, or vascular death) in patients with multivessel disease including ICVD and PAD (15). These findings further highlight the strong association between cerebrovascular disease and peripheral arterial disease.

The total length of the lesion and the duration of the procedure are markers of the complexity of the lesion (16). Our findings suggest that lesion length greater than 150 mm increases the risk of postprocedural CD-TLR and may therefore contribute to poorer outcomes in patients with FPAD. Diehl and Bosiers found restenosis rates of 28.3% and 27.8% after stenting lesions with mean lengths of 9.4 cm and 9.6 cm, respectively (17, 18). Additionally, a prospective study by Krankenberg, which included 989 patients with FPAD, found that for lesions measuring 20 cm, the 12-month restenosis rate increased to 51.2%, further supporting the impact of lesion length on the outcome of superficial femoral artery treatment (19).

In the era of booming endoluminal therapy, there is still a high incidence of restenosis after intervention (20, 21). In recent years, new devices and therapeutic strategies have emerged to overcome this challenge, and drug-coated balloon (DCB) is one of the more promising techniques. The principle of DCB is that the micropores on the surface of the balloon are filled with a drug, mainly paclitaxel, which enters the lesion area through the delivery system of the percutaneous transluminal angioplasty (PTA). Folding the balloon before expansion prevents the drug from being washed away prematurely, and expanding the balloon allows the drug to be immersed into the arterial wall. Some of the drug is washed away by the blood flow when the balloon is released, but the majority remains in the arteries of the localized lesions, thus preventing endothelial hyperplasia (22, 23). After DCB, a new treatment option called atherectomy (ATH) emerged (24). It involves the removal of plaque and blockages in the arteries using specialized equipment, resulting in a more homogeneous angioplasty with minimal trauma to vascular pressure and improved intraluminal gain. ATH reduces the risk of plaque reflection and stripping, and prevents future negative remodeling and intimal hyperplasia, making it widely used in clinical practice (25, 26). However, reviews of ATH have yielded mixed results, and Wardle conducted a systematic evaluation that included seven studies with 527 participants and 581 treated lesions. The evidence regarding the impact of atherosclerotic resection on patency, mortality, and cardiovascular event rates is inconclusive when comparing ATH with balloon angioplasty (BA) and BA combined with stenting, plain balloon angioplasty with or without stenting (27). On the other hand, several recent large randomized controlled trials have shown satisfactory efficacy of ATH combined with DCB compared with DCB alone over a 1-year follow-up period (28–30).

Furthermore, in addition to DCB and ATH, several advanced stent technologies have emerged in recent years. These include drug-eluting stents (DES) and biomimetic stents. DES are medical devices used to treat coronary and peripheral arterial diseases (31). They are coated with drugs that help prevent restenosis and thrombosis, thereby improving the effectiveness of interventional therapy and reducing the need for repeat procedures (32). Biomimetic stents, on the other hand, are typically made from metal alloys or biodegradable materials. They are designed to mimic natural biological structures and functions, withstanding arterial blood flow and pressure to prevent recurrent stenosis (33). A systematic review and meta-analysis focusing on the application of biomimetic stents in infra-inguinal peripheral arterial disease (IPAD) concluded that biomimetic stents have advantages over conventional stents. They were found to reduce restenosis rates and improve vascular patency, possibly due to their enhanced biocompatibility and reduced inflammatory response. Biomimetic stents also showed promising outcomes in reducing thrombosis and complications associated with in-stent restenosis, such as target vessel revascularization. However, the review highlighted the necessity for larger-scale, longer-term, and higher-quality studies to further establish the efficacy of biomimetic stents in IPAD treatment, as well as to determine their optimal conditions and indications for use (34).

Additionally, while our study did not specifically address the impact of gender on adverse events after endovascular treatment for FPAD (femoral-popliteal artery disease), some literature has indeed highlighted this issue. Krishnan conducted an analysis of 195 FPAD patients (93 females and 102 males) who underwent DCB (drug-coated balloon) treatment (35). By comparing the ABI (Ankle-Brachial Index) and peak systolic velocities (PSV) between male and female patients at 6, 12, and 24 months post-intervention, Krishnan found that females had significantly lower ABI and PSV values compared to males, accompanied by a higher TLR (target lesion revascularization) rate.

Regarding the issue of recanalization after CD-TLR (catheter-directed thrombolysis with or without thrombolytic therapy), various techniques are available for revascularization following stenosis. Fresilli evaluated the feasibility and effectiveness of an intravascular ultrasound (IVUS)-guided intra-guide re-entry catheter (IGRC) for the treatment of femoral-popliteal chronic total occlusions (FP-CTO), comparing it to a bidirectional approach without IGRC (36). The study revealed that while the success rate of FP-CTO recanalization was comparable between the IGRC and bidirectional methods, the use of IGRC reduced radiation exposure, the amount of iodinated contrast agent, patient discomfort, and operative time. These advantages suggest that IGRC could serve as a viable next option after failed retrograde recanalization in FP-CTO cases.

Our study demonstrated that the use of ATH or DCB reduced the incidence of 1-year CD-TLR in FPAD patients, which is consistent with the results of the aforementioned trials. Further studies are needed to validate the role of these factors in FPAD. The developed nomogram offers several clinical implications. Firstly, it provides a quantitative tool for clinicians to more accurately predict 1 year CD-TLR of FPAD than traditional methods, aiding in better risk stratification. Moreover, early identification of high-risk individuals through this nomogram can lead to timely interventions, potentially reducing morbidity and mortality.

This study has several limitations that should be acknowledged. The cohort was based on retrospectively collected patient data, which may not be representative of the wider population. Furthermore, there may be potential unmeasured confounders that were not included in our model. External validation in diverse populations will be essential to confirm the generalizability of our findings.

Future research should aim to externally validate our nomogram in different populations and settings. Additionally, integrating novel predictors or biomarkers could enhance the predictive accuracy of the nomogram, warranting further investigation.

## Conclusions

The newly developed predictive model can accurately predict clinically driven target revascularization events in FPAD patients at 1 year, providing valuable information for the development of individualized treatment plans.

## Data Availability

The raw data supporting the conclusions of this article will be made available by the authors, without undue reservation.
