# Supplementary material for: Innovative nomogram for predicting 1-year clinically driven revascularization outcomes in endovascular femoropopliteal disease
Source: Front Cardiovasc Med. 2024 Aug 28;11:1438214. doi: 10.3389/fcvm.2024.1438214 (PMC11387798; doi:10.3389/fcvm.2024.1438214)
Supplement: Supplementary file 1 [file Datasheet1.docx]

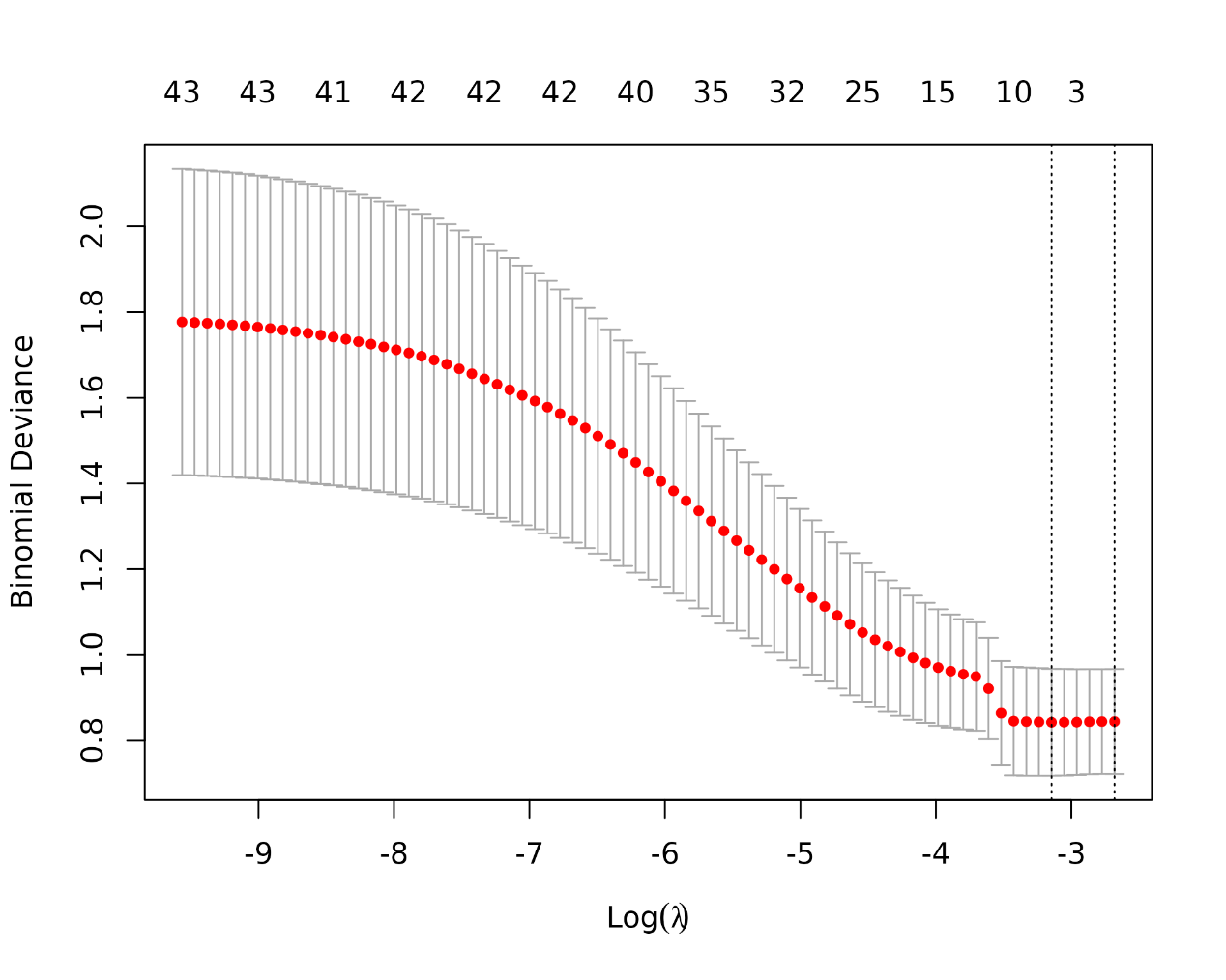


Figure S1 Lasso Regression Cross-Validation Plot


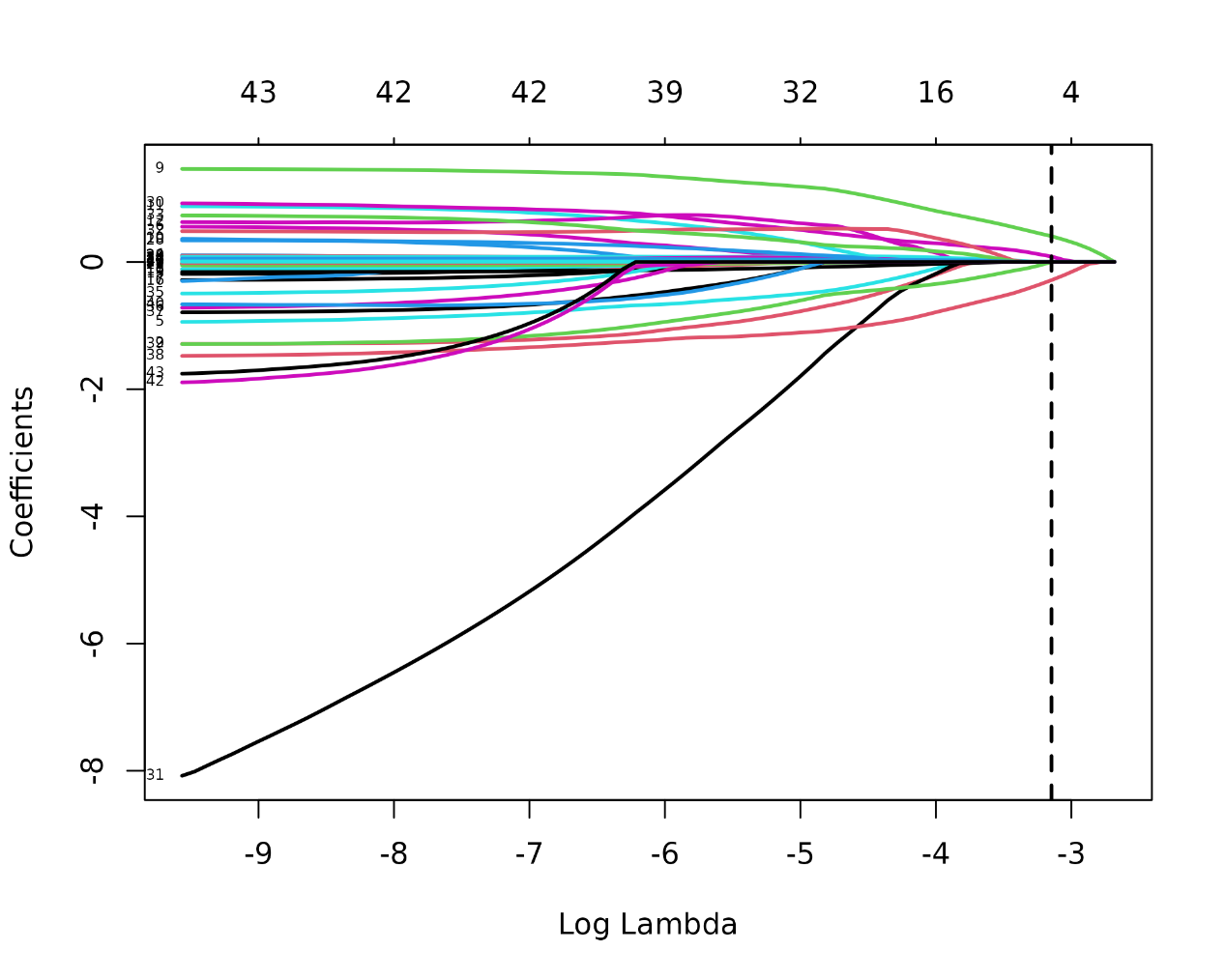


Figure S2 Lasso Regression Coefficient Path Plot
